# The Effect of Alexithymia on Attentional Bias Toward Emotional Stimuli in Depression: An Eye-Tracking Study

**DOI:** 10.3389/fpsyt.2020.569946

**Published:** 2021-02-17

**Authors:** M. Annemiek Bergman, Constance Th. W. M. Vissers, Rose M. Collard, Philip van Eijndhoven, Aart H. Schene, Janna N. Vrijsen

**Affiliations:** ^1^Department of Psychiatry, Radboud University Medical Centre, Nijmegen, Netherlands; ^2^Donders Institute for Brain, Cognition and Behavior, Donders Center for Medical Neuroscience, Nijmegen, Netherlands; ^3^Royal Dutch Kentalis, Kentalis Academy, Sint-Michielsgestel, Netherlands; ^4^Radboud University Nijmegen, Behavioural Science Institute, Nijmegen, Netherlands; ^5^Pro Persona Mental Health Care, Depression Expertise Center, Nijmegen, Netherlands

**Keywords:** alexithymia, depression, attentional bias, executive functioning, emotion regulation

## Abstract

Alexithymia—reflecting deficits in cognitive emotion processing—is highly prevalent in individuals with depressive disorders. Subsequently, mixed evidence for attentional bias is found in these individuals. Alexithymia may be a potential influencing factor for attentional bias in depression. In the current study, 83 currently depressed (CD) and 76 never-depressed (ND) controls completed an eye-tracker task consisting of valenced (non)-social pictures. Alexithymia scores were also included as a moderator as both a continuous and categorical measure (so high vs. low alexithymia). No group difference or moderating effect of alexithymia was found on attentional bias. Thus, alexithymic symptoms, included both dimensionally and categorically, may not influence biased attentional processing in depression compared to ND individuals. Thus, it is important to explore other potential explaining factors for the equivocal results found on biased attentional processing of emotional information in depression.

## Introduction

Alexithymia reflects a deficit in the cognitive processing of emotions (1). Alexithymia has two components. The emotional component is a reduced ability to identify, describe, and express emotional feelings of oneself and others, as well as describing somatic experiences. On the other hand, the cognitive component of alexithymia is reflected by poor introspective thinking that is considered to be an externally oriented cognitive style (2, 3).

In the general population, 8–13% of persons show alexithymic traits (4–6). Moreover, these traits are more prevalent across a wide range of somatic and psychiatric disorders (7–10) and are particularly high (26–50%) among patients with depressive disorders (9, 11). Depressed patients with alexithymia show more severe depressive symptoms and more overall psychopathology compared to depressed non-alexithymic patients (12). Deficits in identifying and recognizing emotional feelings due to co-occurring alexithymia could lead to misinterpretation of depressive symptoms as symptoms of a somatic disorder. It could also result in depressive symptoms staying undetected and, thus, possibly develop into more severe or chronic depressive symptoms (13). This increase of depressive symptoms that may be due to comorbid alexithymia underscores the need to investigate possible factors that could play a role in the strong relation between alexithymic symptoms and depression.

Since individuals with alexithymia experience deficits in recognizing and describing internal and external emotional feelings, the effects of alexithymia can be reflected in the person's cognitive (automatic) processing of emotional information (1). This implies that emotional cues are less attended and, thus, alexithymia may result in reduced attentional bias for emotional information compared to neutral information. Hence, these individuals may allocate less attention toward (i.e., they spent less time looking at) emotional stimuli (i.e., negative or positive) relative to neutral stimuli.

In the few studies that focus on the association between alexithymia and cognitive biases for emotional stimuli in community or student samples, strong indications are observed for a stronger association between high levels of alexithymia and less cognitive bias for negative stimuli [e.g., (14–17)] or less attentional bias toward dysphoric (e.g., sad, hopelessness) stimuli in highly external orienting alexithymic individuals (18). Conversely, others did not find an association of alexithymia and bias for negative stimuli in their entire community sample (19) or demonstrated decreased processing of positive stimuli in high alexithymic (HA) relative to low alexithymic (LA) individuals (20). Hence, evidence exists that high levels of alexithymia may possibly influence biased information processing of emotional stimuli. Nevertheless, this might be specific for negative information (i.e., threatening or dysphoric). Thus far, most studies investigated the effect of alexithymia on biased information processing in non-clinical samples. However, cognitive biases for especially negative information are present in diverse clinical populations (e.g., anxiety disorders) but are most prominently found in depressive disorders (21).

A leading cognitive model of depression posits a mood-congruent bias in which negative affect provokes enhanced attention toward negative stimuli (22, 23). Such a bias may stem from adverse childhood experiences resulting in the development of dysfunctional cognitive schemata, which can form the base for a cognitive vulnerability for developing depression. The empirical findings for negative attentional bias in (sub)clinically depressed individuals are equivocal (24, 25). Studies have demonstrated more negative (i.e., allocating more attention toward negative compared to positive and neutral stimuli) as well as less positive attentional bias (i.e., attending less to positive information compared to negative information) in currently depressed (CD) individuals compared to non-depressed individuals [e.g., (21, 26)]. Thus, depressed individuals tend to focus more on negative material than non-depressed individuals. However, lack of associations of negative attentional bias with (sub)clinical depression has also been reported (27–29). Considering inconclusive evidence is found for negative attentional processing of information in depression, examining possible influencing factors on this relationship is necessary. This is important because negative attentional bias is considered to be a risk factor in the development, maintenance, and recurrence of (symptoms of) depression [e.g., (30, 31)].

The current study will investigate whether alexithymia influences attentional bias in depression. To this aim, CD and never-depressed (ND) individuals were included in the study, and a free-viewing eye-tracking task was used, allowing the measurement of preferential attentional allocation toward valenced stimuli (i.e., attentional bias). Free-viewing eye-tracking paradigms have been established to have good reliability for measuring attentional bias (32). In depression, attentional bias is associated with a general negative bias (33). Thus, in the current study, general negatively and positively valenced pictures (e.g., a family hugging vs. a couple crying; a litter of kittens vs. a neglected dog) were included. Further, to assess alexithymia, the 20-item self-report Toronto Alexithymia Scale [TAS-20 (34)] was used, which is the most commonly used questionnaire for assessing alexithymia. Important to note is that differences in the inclusion of alexithymia symptom scores in studies exist: Both a dimensional and a categorical approach in which a cutoff score (i.e., by using a median split, extreme scores, or the clinical cutoff scores) is used in research (35, 36). Therefore, in the current study, we will include both a categorical (i.e., using extreme groups representing the top and bottom 33% of alexithymia in the sample) and dimensional approach (i.e., using a continuous measure of alexithymia) to examine whether alexithymia influences the presence of negative attentional bias in depression.

We hypothesized that the depressed individuals would show more negative relative to positive attentional bias compared to ND individuals. In addition, based on previous findings in HA ND individuals, we expect HA depressed patients and ND controls to show less negative relative to positive attentional bias compared to LA depressed patients. Put differently, we expected the HA depressed individuals to resemble the ND controls. Additionally and fully in line with this, we expected the continuous measure of alexithymia to moderate the group effect (depressed vs. controls) on attentional bias. If substantiating evidence is found, this could indicate that the presence of co-occurring alexithymia may partially account for the mixed results found in the attentional bias literature for depression. Since discrepancy exists in the current literature in the approach of including alexithymic symptoms (i.e., categorical vs. dimensional), we will additionally explore if alexithymic symptoms, as measured with the total scores of the TAS-20, could be a possible moderator on attentional bias in patients with a current depressive disorder and ND controls.

## Materials and Methods

### Participants

This study is part of the ongoing cross-sectional MIND-Set study (Measuring Integrated Novel Dimensions in Neurodevelopmental and Stress-related mental disorders) at the Department of Psychiatry of the Radboud University Medical Center (Radboudumc), Nijmegen, the Netherlands. Adult psychiatric patients (18 and older) with at least one clinical diagnosis of a stress-related disorder (depression, anxiety, and/or substance use disorders) and/or neurodevelopmental disorder [autism spectrum disorder (ASD) and/or attention deficit/hyperactivity disorder (ADHD)] were eligible to participate. The ND controls were recruited by promotion of the study in the community (e.g., social media, flyers, and websites) *via* the Radboud Research Participation System as well as verbally through researchers' own networks. The ND received a small fee for taking part in the study. Participants with current psychosis, sensorimotor handicaps, epilepsy (only for the eye-tracker task), inadequate command of the Dutch language, a full-scale IQ estimate of below 70, and/or mental incompetence to sign the informed consent form were excluded from participation. All participants had normal or corrected-to-normal vision. The study has been approved by the Ethical Review Board of the Radboudumc, and all participants signed an informed consent prior to participating in this study. Patients with a diagnosis of a current depressive disorder [i.e., major depressive disorder (MDD) and/or dysthymic disorder; CD] and ND were included as an analytical subsample of the MIND-Set study in the current paper, given that the diagnostic and demographic measures were fully assessed, and data of the eye-tracking task were complete. The data of this subsample were collected between August 2016 and July 2019. Eye-tracking data of nine CD and four ND were excluded due to poor calibration. A total of 83 CD and 76 ND were included in the final sample of this study. See Table 1 for the demographic variables of all the participants.

**Table 1:** Group comparisons of the final sample on demographic variables and TAS-20 (total and subscale) scores [means and standard deviations (*SD*)] including test statistics for the group comparisons.

**Variable**	**CD (*n* = 83)**	**ND (*n*= 76)**	**Group comparisons**
**Group**			
IDS-SR	40.0 (11.95)	4.9 (4.1)	*F*_(1, 149)_ = 559.53, *p* < 0.001
TAS-20	56.6 (12.32)	38.7 (6.3)	*F*_(1, 157)_ = 102.05, *p* < 0.001
Gender, female (%)	42.6	54.7	$\chi_{\left(1\right)}^{2}$ = 4.50, *p* = 0.034
Age, mean (*SD*)	40.5 (14.87)	38.29 (15.99)	*F*_(1, 157)_ = 0.82, *p* = 0.367
Education level^a^			$\chi_{\left(2\right)}^{2}$ = 6.41, *p* = 0.041
Low (%)	17.8	5.8	
Middle (%)	33.7	37.2	
High (%)	42.6	57.0	

*CD, currently depressed patients; ND, never depressed controls; TAS-20, Toronto Alexithymia Scale*.

*TAS-20 DIF, difficulty identifying feelings; TAS-20 DDF, difficulty describing feelings; TAS-20 EOT, externally oriented thinking; IDS-SR, Inventory of Depressive Symptomatology (self-report)*.

a *Adjusted classification of Ikram et al. (37). Low, no education or elementary education and lower vocational and general secondary education were combined; Middle, intermediate vocational and higher secondary education; High, higher vocational education or university*.

#### Diagnostic Procedure

To classify the depressive patients (and also for the possible presence of comorbid psychiatric disorders) and to check for the absence of a lifetime history of psychiatric disorders in the ND participants, the following diagnostic screeners as well as semistructured interviews were used for all participants. The diagnostics were carried out face-to-face during the transition period from Diagnostic and Statistical Manual of Mental Disorders (DSM)-IV to DSM-5 at the Radboud University Medical Center, Psychiatry Department. The ND participants were screened *via* telephone. Depressive disorders, anxiety disorders, substance use disorders, and ADHDs were diagnosed according to DSM-IV; ASDs, according to DSM-5 criteria. Depressive and anxiety disorders were diagnosed with the Structured Clinical Interview for DSM-IV Axis I Disorders [SCID-I; (38)] and substance use disorders with the Measurements in the Additions for Triage and Evaluation and Criminality [MATE-Crimi; (39)]. To diagnose ADHD, we used the interview for ADHD in Adults Version 2.0 [Dutch: Diagnostisch Interview voor ADHD bij volwassenen 2.0; DIVA 2.0 (40, 41)] and, for ASD, the Dutch Interview for Diagnosing Autism Spectrum Disorders [in Dutch: “Nederlands Interview ten behoeve van Diagnostiek Autismespectrumstoornissen” (NIDA); (42)] was used. See Table 2 for the comorbid disorders present in the CD patients.

**Table 2:** Prevalence of comorbid psychiatric disorders in the CD patient group.

**Comorbid disorder**	**CD (*n* = 83)**
**Group**	
ADHD (%)	22
Anxiety disorder (%)	23
ASD (%)	21
Substance use disorder (%)	19
Number of comorbid disorders (%)	1 = 47, 2 = 13, 3 = 4

*CD, currently depressed patients; ADHD, attention deficit/hyperactivity disorder; ASD, autism spectrum disorder*.

All participants in this study completed online questionnaires to assess depressive symptom severity and alexithymia. Symptom severity of depression was measured with the 30-item Inventory of Depressive Symptomatology self-report version [IDS-SR; (43)]. Total scores ranging from 0 to 84, scores ≤13 are considered within the normal range; scores of 14–21 indicate mild depression; 22–38 moderate MDD; ≥39 severe depression. The IDS-SR has good psychometric properties and internal consistency (43). The TAS-20 (44) was used to assess alexithymia (range 20–100). Higher scores on the TAS-20 indicate higher levels of alexithymia. The TAS-20 has good reliability and validity (34, 44).

### Materials and Apparatus

#### Free-Viewing Task

**Figure 1:**
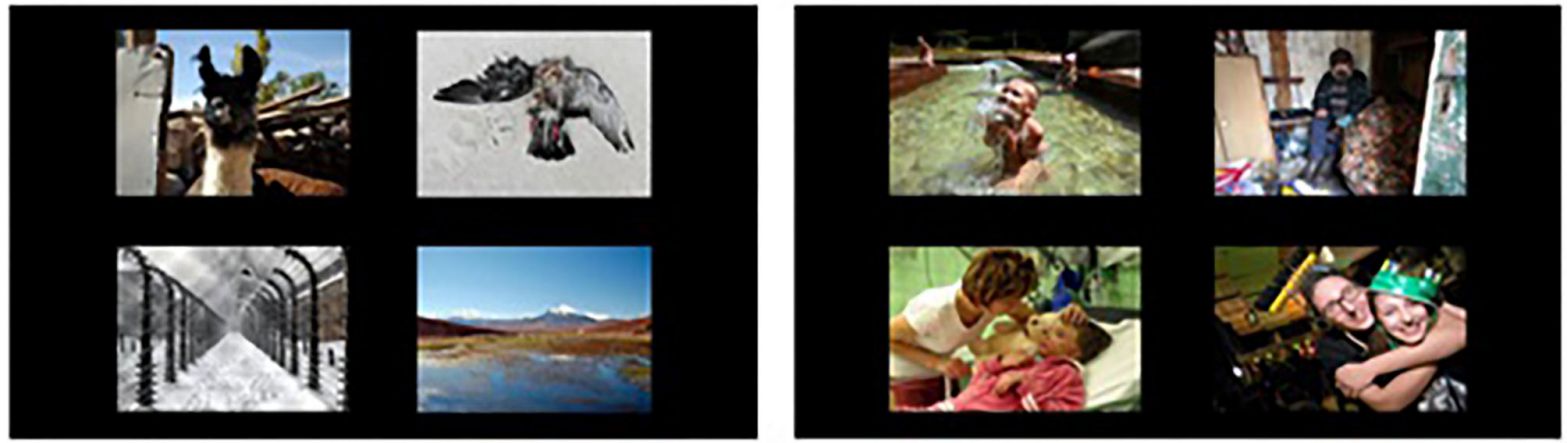
**Left:** an example slide from the non-social block; **Right:** an example slide from the social block. All stimuli in this study, including the ones depicted in these example slides, have been obtained from two public databases, namely, the Nencki Affective Picture System (47) and the International Affective Picture System (46).

The free-viewing eye-tracking task implemented in this study is similar to the task used in Bergman et al. (45). The stimuli included a total of 96 pictures selected from the International Affective Picture System [IAPS; (46)] and the Nencki Affective Picture System [NAPS; (47)]. One half of the pictures displayed were negatively or positively valenced social pictures (i.e., a hugging family; a crying woman); the other half negatively or positively valenced non-social pictures (i.e., a neglected dog; a litter of kittens). Thus, the 24 trials contained four images on each slide either of the social or non-social category, with the constraint that two pictures of the same valence were not presented next to each other. The two blocks (i.e., one block was composed of all the social and one block of all the non-social stimuli) were presented in a random order across the participants. For the purpose of this study, the valenced social and non-social pictures were compiled into two categories of general positive or general negative stimuli. See Figure 1 for an example of a social and a non-social slide.

#### Eye Tracker

A screen-based eye-tracking system (SMI RED500) was employed to measure the participants' eye movements, allowing a free range of head movements. The direction of gaze within the screen, measured with x and y coordinates, was sampled every 2 ms (500 Hz). Data were collected using a velocity-based algorithm with a minimum fixation duration threshold of 100 ms and a peak velocity threshold of 40°/s. Areas of interest (AOIs) were also identified for each slide and corresponded with the total area for each of the four images including the outer corners to include possible recording noise. A total of two AOIs were constructed: a positive and a negative one. The eye movement data were preprocessed with the SMI BeGaze software version 3.7 (SensoMotoric Instruments, Inc., Teltow, Germany). In addition, the data were visually inspected for abnormalities and checked for distributional anomalies, which were not found.

#### Relative Gaze Duration

The relative gaze duration (i.e., the sum of time participants looked at negative or positive stimuli relative to the total gaze duration) was computed as the sum of time participants looked at negative or positive stimuli divided by the total time the particpants spent looking at the screen. Subsequently, the negative relative gaze duration was subtracted from the positive relative gaze duration resulting in one relative gaze duration difference score [i.e., relative attentional bias score (ABS)] per participant. This ABS was used in all analyses (i.e., the one-way ANCOVAs and the moderation analysis). Negative values for relative ABS indicated a negative bias (i.e., individuals gazed longer at negative compared to positive stimuli), whereas positive relative ABS values reflected a positive bias (i.e., individuals gazed longer at positive compared to negative stimuli).

### Procedure

A participant was seated in a height-adjustable chair approximately 60 cm in front of a 22″ Dell TFT-monitor on which the stimuli were presented. The experimental task started when the 9-point calibration procedure was successfully completed [i.e., the average error was a maximum of 1.5° of the visual angle for the calibration points; in line with (48)]. This calibration procedure was rerun in between every block. All participants were instructed to minimize head movements, avoid speaking, and to look freely at the pictures on the screen. The eye-tracking task consisted of two parts, which the participant was instructed about beforehand, the free-viewing task described before and the recognition task. The recognition task was included to encourage active viewing of the pictures and mask the purpose of the free-viewing task. During the recognition task, two out of four pictures of the previously presented slides have switched positions and the participant was asked to indicate which one out of these two. The total duration of the whole eye-tracking procedure was approximately 20 min.

### Statistical Analysis

Firstly, we wanted to examine if the depressed individuals differed from the ND controls on ABS. Thus, a one-way between-subjects ANCOVA analysis was conducted with Group (CD and ND) as the independent variable and relative ABS as the dependent variable. Age, gender, and education level were included as covariates. Next, a separate analysis was conducted to investigate if depressed patients with HA symptoms differed from patients with LA symptoms and ND controls on ABS. Therefore, we divided the patient sample into two groups: LA (lowest 33%, TAS-20 < 54) CD (LA-CD), HA (highest 33%, TAS-20 > 63) CD (HA-CD) and compared them to the ND controls. To ensure that the ND and LA-CD groups were equally distributed on the alexithymia scores, we omitted ND with a TAS-20 score of 54 or more (*n* = 9), which formed the new ND group (ND-A) included in this specific analysis. In this second ANCOVA analysis, Group (LA-CD, HA-CD, and ND-A) was included as the independent variable. Relative ABS was the dependent variable, and age, gender, and education level were included as covariates.

**Figure 2:**
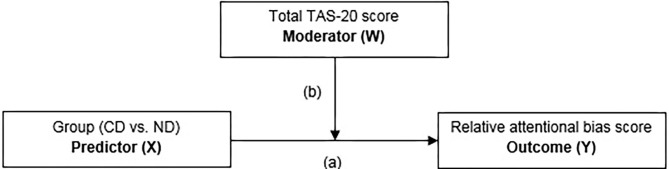
Moderation model with group [currently depressed (CD) individuals vs. never depressed (ND) controls] as predictor (X), total TAS-20 score as the moderator (W), and the relative attentional bias score as the outcome (Y) variable.

In the moderation analysis, the TAS-20 total score was investigated as a moderator (W) on the group (i.e., all CD and ND individuals; X) and relative attentional bias score (relative ABS; Y) relationship using the PROCESS 3.4 macro tool for SPSS (49). The PROCESS macro tool examines the outcome of (X), the proposed moderator (W), and the interaction of the two. There is evidence for moderation when the 95% confidence interval does not include zero (50), 1,000 bootstrap samples were performed. See Figure 2 for the specific details of the moderation model.

## Results

### Group Comparisons on Symptoms and Demographics

The CD and ND group did not differ significantly with respect to age. However they differed significantly on TAS-20 total scores, depressive symptoms, education level, and gender identification (Table 1).

### The Effect of High and Low Alexithymia in Depressed vs. Never-Depressed Groups on Attentional Bias

**Figure 3:**
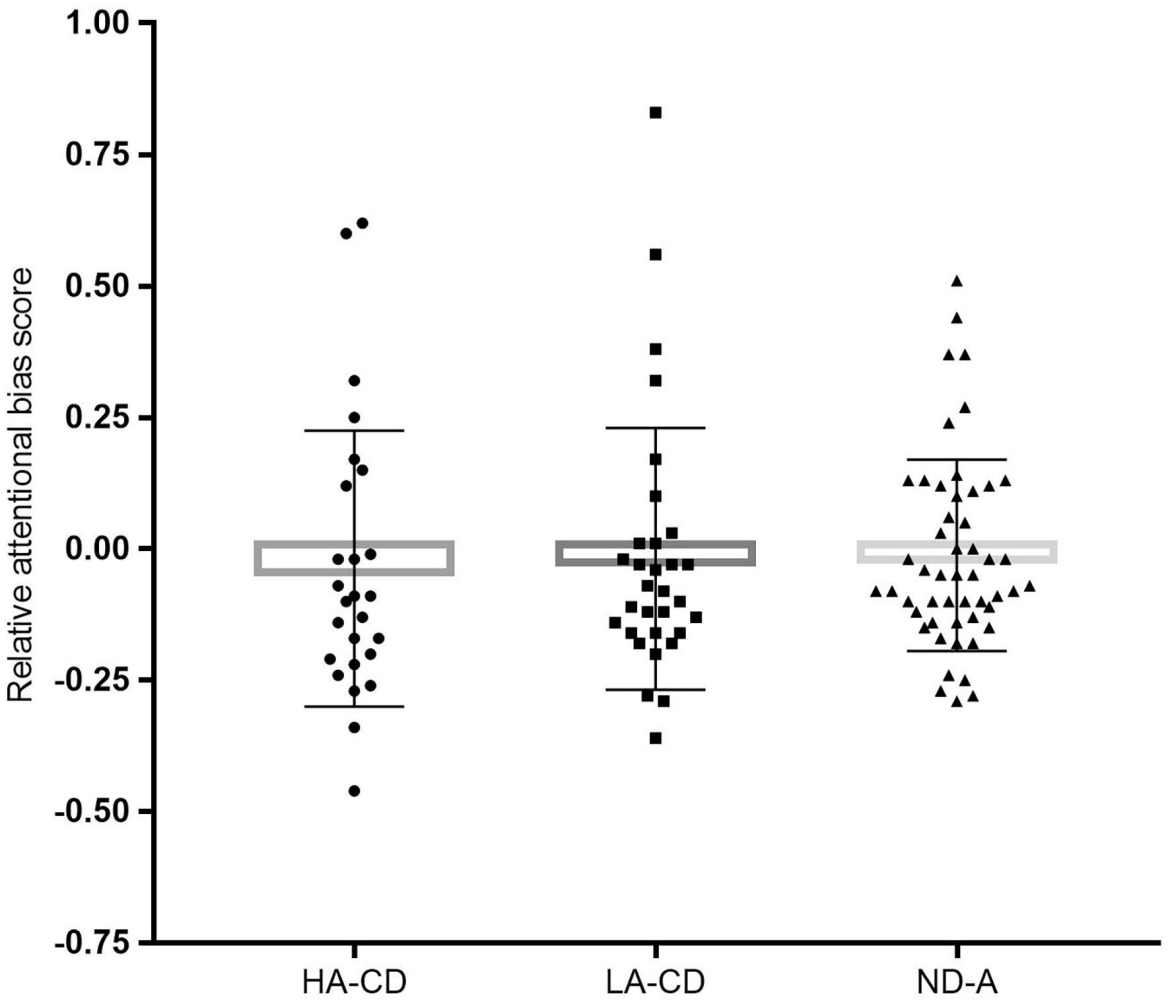
The graph represents the mean relative attentional bias scores for each group with individual data points: HA-CD, high alexithymia currently depressed (*n* = 29); LA-CD, low alexithymia currently depressed (*n* = 28); ND-A, never depressed controls (*n* = 47). Error bars represent standard deviations.

CD individuals (*M* = −0.012, *SD* = 0.257) and ND controls (*M* = −0.028, *SD* = 0.173) did not differ significantly on relative ABS, *F*_(1, 158)_ = 0.33, *p* = 0.564. The one-way between-subjects ANCOVA including differentiation on alexithymia level was aimed to investigate if alexithymia level obscured group differences on attentional bias. Therefore, the following groups were compared: CD patient with high (top 33%) alexithymia levels (HA-CD, *n* = 29), CD with low level of alexithymia (bottom 33%; LA-CD, *n* = 28), and ND individuals with low level of alexithymia (ND-A, *n* = 47). However, Group did not show a significant difference in relative ABS, *F*_(2, 100)_ = 0.05, *p* = 0.950; see also Figure 3 for more details. Thus, the groups (i.e., HA-CD, LA-CD, and ND-A) showed comparable attentional biased processing of valenced stimuli. Because the 95% CI of the group comparison including the alexithymia subdivision (i.e., comparing LA-CD, HA-CD, and ND-A) includes zero (see also Table 3), we cannot reject the null hypothesis of no group differences.

**Table 3:** The means (*M*) with standard deviations (*SD*s) and adjusted means (*M*_adj_) with standard errors (*SE*s) of the one-way between-subjects ANCOVA comparing the relative attentional bias score of the depressed individuals with high or low alexithymic symptoms and never-depressed controls on the relative attentional bias score with the corresponding 95% confidence intervals.

**Group**	***M***	***SD***	***M*_**adj**_**	***SE***	**95% CI**
HA-CD	−0.038	0.262	−0.038	−0.044	[−0.126, 0.050]
LA-CD	−0.019	0.248	−0.021	−0.038	[−0.096, 0.055]
ND-A	−0.012	0.182	−0.021	−0.030	[−0.080, 0.037]

*Group: high alexithymia (HA-CD) depression patients, low alexithymia (LA-CD) depression patients, and never-depressed controls (ND-A). Means (M), standard deviations (SDs), adjusted means (M_adj_), standard errors (SEs), and 95% confidence intervals (CIs) for relative attentional bias score (ABS) for the three groups*.

### The Moderating Effect of Alexithymia on Attentional Bias

To assess if TAS-20 scores moderated the relationship of CD and ND groups and the relative ABS, a moderation analysis was conducted; see Figure 2 for the specific details of the moderation model. No main or interaction effects were significant (*p* > 0.232); see Table 4 for the *t*-statistics of the moderation analysis. Important to note is that the groups did not significantly differ on ABS (*p* = 0.231), indicating no differential bias dependent on depression status. Further, no moderation effect of alexithymia on ABS was found (*p* = 0.655). Because the 95% CI of the interaction effect of Group^*^TAS-20 score includes zero, see also Table 4, we cannot reject the null hypothesis of no group differences.

**Table 4:** Moderation of alexithymia on the effect of group on relative attentional bias score (ABS).

	***b***	**95% CI**	***SE B***	***t***	***p*-value**
Constant	0.04	[−0.14, 0.21]	0.09	0.40	0.691
Group	−0.05	[−0.13, 0.03]	0.04	−1.20	0.231
TAS-20 score (centered)	−0.01	[−0.01, 0.01]	0.01	−0.97	0.337
Group × TAS-20 score	0.01	[−0.01, 0.01]	0.00	−0.45	0.655
Gender	0.11	[0.04, 0.18]	0.03	3.18	0.002^*^
Age	0.001	[−0.01, 0.01]	0.01	0.48	0.632
Education level	−0.03	[−0.08, 0.01]	0.02	−1.39	0.166

*R^2^ = 0.08. Group includes currently depressed and never depressed controls. Standard error (SE) and 95% confidence interval (CI)*.

* *p < 0.05*.

## Discussion

In the current study, we aimed to examine whether alexithymia could influence the relationship between depression and biased attentional processing of valenced information. If alexithymia indeed affects attentional bias processing in depressed individuals, then this might partially explain the mixed results in attentional bias research in depression. First, the present study found no differences in attentional bias for valenced emotional stimuli in CD and ND controls. This result is pursuant to others demonstrating no differential attentional bias processing in these groups [e.g., (27, 29, 51)]. In contrast, others did demonstrate more negative as well as less positive attentional bias (21, 26). The present study adds to the growing body of research suggesting that attentional bias may not be a robust marker for (symptoms of) depressive disorders [e.g., (52)].

Second, alexithymia did not moderate the depressed group differences on attentional bias for positive and negative stimuli. These results corroborate the study of Lundh and Simonsson-Sarnecki (19), who found no differences in attentional bias when investigating the entire participant sample, which was a community-based sample. However, a *post hoc* power analysis revealed that in order to detect a significant effect at 5% with a statistical power at the recommended 0.80 level (53), a sample of 196 participants would be required. Future studies with larger sample sizes are thus needed to detect a possible influence of alexithymic symptoms on biased attentional processing in depressed individuals. Taken together, these findings suggest that alexithymic symptoms may not influence biased attentional processing in depression. However, considering the limited studies available, further research is needed to confirm these findings.

In the current study, a direct effect of alexithymia on biased attentional processing was not demonstrated. Possibly, this might be due to an indirect influence of alexithymia on attentional bias by a moderating effect of, for instance, deficits in executive functioning (EF). EF is interrelated with other cognitive processes such as attentional bias (54, 55) and alexithymia (56–59). Deficits in EF are also connected with depressive disorders [see (60, 61)]. An aspect of the self-regulatory action of “self-directed emotion/motivation” of Barkley's EF theory is emotion regulation. This EF represents the inhibition of strong emotions and to downregulate or otherwise moderate them (62, 63). Research confirms that alexithymia is associated with poor emotion regulation strategies [e.g., (64)]. Specifically, suppression (i.e., to inhibit an emotional response) has been found to decrease positive and not negative emotion experience. Future studies could advance our knowledge of this interplay by including (self-report) measures of executive functioning, specifically for emotion regulation.

Strength of this study is the inclusion of a naturalistic, well-defined patient sample, which facilitates the generalizability of the current findings to the clinical population. Another strength is the use of an eye tracker, which is proven to be a reliable measure to assess attentional bias toward valenced stimuli in depressed individuals, especially compared to more traditional measures such as a the dot-probe and emotional Stroop tasks (31, 32). In addition, we examined both approaches (i.e., categorical and dimensional) for including the TAS-20 measure in our study, thus, to thoroughly investigate a potential effect of alexithymic symptoms on attentional bias. It is important to note that the TAS-20 solely relies on self-assessment, which can pose as a problem since it is shown that alexithymic individuals have a poor general ability of self-reflection (65). Nevertheless, the TAS-20 has been proven to be a valid measure of alexithymia in several common psychiatric disorders, and it has good discriminative validity between healthy and clinical individuals (1).

In conclusion, we did not find evidence for a negative attentional bias in depressed patients, contributing to the mixed findings on attentional bias in depression. Moreover, alexithymia did not seem to explain the lack of negative bias in depression in our sample. Thus, it would be of importance to explore other potential explaining factors for the equivocal results found on biased attentional processing of emotional information in depression, such as the influence of psychiatric comorbidity (e.g., anxiety disorders, ASDs) or individual differences on emotion processing aspects (e.g., emotion regulation, anhedonia).

## Data Availability Statement

The raw data supporting the conclusions of this article can be made available via a request directed at the coordinator (Janna N. Vrijsen) of the MIND-Set study. Requests to access the datasets should be directed to janna.vrijsen@radboudumc.nl.

## Ethics Statement

The studies involving human participants were reviewed and approved by Commissie Mensgebonden Onderzoek regio Arnhem–Nijmegen (WMO). The patients/participants provided their written informed consent to participate in this study.

## Author Contributions

All authors listed have made a substantial, direct and intellectual contribution to the work, and approved it for publication.

## Conflict of Interest

The authors declare that the research was conducted in the absence of any commercial or financial relationships that could be construed as a potential conflict of interest.
